# Phylogeny and Phylogeography of a Recent HIV-1 Subtype F Outbreak among Men Who Have Sex with Men in Spain Deriving from a Cluster with a Wide Geographic Circulation in Western Europe

**DOI:** 10.1371/journal.pone.0143325

**Published:** 2015-11-24

**Authors:** Elena Delgado, María Teresa Cuevas, Francisco Domínguez, Yolanda Vega, Marina Cabello, Aurora Fernández-García, Marcos Pérez-Losada, María Ángeles Castro, Vanessa Montero, Mónica Sánchez, Ana Mariño, Hortensia Álvarez, Patricia Ordóñez, Antonio Ocampo, Celia Miralles, Sonia Pérez-Castro, María José López-Álvarez, Raúl Rodríguez, Matilde Trigo, Julio Diz-Arén, Carmen Hinojosa, Pablo Bachiller, Silvia Hernáez-Crespo, Ramón Cisterna, Eugenio Garduño, Lucía Pérez-Álvarez, Michael M Thomson

**Affiliations:** 1 HIV Biology and Variability Unit, Centro Nacional de Microbiología, Instituto de Salud Carlos III, Majadahonda, Madrid, Spain; 2 Centro de Investigação em Biodiversidade e Recursos Genéticos (CIBIO-InBIO), Vairão, Portugal; 3 Department of Internal Medicine, Complejo Hospitalario Universitario de A Coruña, A Coruña, Spain; 4 Infectious Diseases Unit, Department of Internal Medicine, Complejo Hospitalario Universitario Arquitecto Marcide, Ferrol, A Coruña, Spain; 5 Department of Microbiology, Complejo Hospitalario Universitario Arquitecto Marcide, Ferrol, A Coruña, Spain; 6 Department of Internal Medicine, Complejo Hospitalario Universitario de Vigo, Vigo, Pontevedra, Spain; 7 Department of Microbiology, Complejo Hospitalario Universitario de Vigo, Vigo, Pontevedra, Spain; 8 Infectious Disesases Unit, Hospital Universitario Lucus Augusti, Lugo, Spain; 9 Department of Internal Medicine, Complejo Hospitalario Universitario de Ourense, Ourense, Spain; 10 Department of Microbiology, Complejo Hospitalario Provincial de Pontevedra, Pontevedra, Spain; 11 Department of Internal Medicine, Complejo Hospitalario Provincial de Pontevedra, Pontevedra, Spain; 12 Department of Internal Medicine, Hospital Clínico Universitario de Valladolid, Valladolid, Spain; 13 Department of Internal Medicine, Hospital Universitario Río Hortega, Valladolid, Spain; 14 Department of Clinical Microbiology and Infection Control, Hospital Universitario de Basurto, Bilbao, Vizcaya, Spain; 15 Department of Microbiology, Hospital Infanta Cristina, Badajoz, Spain; Institut Pasteur of Shanghai,Chinese Academy of Sciences, CHINA

## Abstract

We recently reported the rapid expansion of an HIV-1 subtype F cluster among men who have sex with men (MSM) in the region of Galicia, Northwest Spain. Here we update this outbreak, analyze near full-length genomes, determine phylogenetic relationships, and estimate its origin. For this study, we used sequences of HIV-1 protease-reverse transcriptase and *env* V3 region, and for 17 samples, near full-length genome sequences were obtained. Phylogenetic analyses were performed via maximum likelihood. Locations and times of most recent common ancestors were estimated using Bayesian inference. Among samples analyzed by us, 100 HIV-1 F1 subsubtype infections of monophyletic origin were diagnosed in Spain, including 88 in Galicia and 12 in four other regions. Most viruses (n = 90) grouped in a subcluster (Galician subcluster), while 7 from Valladolid (Central Spain) grouped in another subcluster. At least 94 individuals were sexually-infected males and at least 71 were MSM. Seventeen near full-length genomes were uniformly of F1 subsubtype. Through similarity searches and phylogenetic analyses, we identified 18 viruses from four other Western European countries [Switzerland (n = 8), Belgium (n = 5), France (n = 3), and United Kingdom (n = 2)] and one from Brazil, from samples collected in 2005–2011, which branched within the subtype F cluster, outside of both Spanish subclusters, most of them corresponding to recently infected individuals. The most probable geographic origin and age of the Galician subcluster was Ferrol, Northwest Galicia, around 2007, while the Western European cluster probably emerged in Switzerland around 2002. In conclusion, a recently expanded HIV-1 subtype F cluster, the largest non-subtype B cluster reported in Western Europe, continues to spread among MSM in Spain; this cluster is part of a larger cluster with a wide geographic circulation in diverse Western European countries.

## Introduction

The HIV-1 epidemic among men who have sex with men (MSM) has experienced a notable upsurge in recent years in many countries [1] associated with increased high risk behavior in this population [1,2]. This has been frequently accompanied by the emergence of local HIV-1 transmission clusters [3–16], whose expansion is mostly driven by onward transmission from individuals with recent infection who are unaware of their HIV status [3,4,8,10–13]. In Western Europe and North America, HIV-1 clusters associated with MSM are usually of subtype B, the clade initially introduced and largely predominant in this population [17]. However, several non-subtype B clusters associated with HIV-1 transmission among MSM have been recently reported in Western countries [14,18–21]. The largest one is an F1 subsubtype cluster of Brazilian ancestry which has rapidly spread among MSM in the region of Galicia, Northwest Spain [21]. Since the original report, covering samples collected up to April 2011, the size of this cluster has increased considerably and its geographic range has expanded. Here we update the information on this cluster, analyze near full-length genomes, determine phylogenetic relationships with viruses from other countries, and estimate its geographic and temporal origin.

## Materials and Methods

### Samples

Plasma samples were collected from 2009 to 2013 from HIV-1-infected individuals residing in all four Galician provinces attended at public hospitals in the cities of A Coruña, Ferrol, Vigo, Pontevedra, Lugo, and Ourense, as well as from individuals attended at 5 hospitals located in four cities of four other Spanish regions: Valladolid (Castilla y León, Central Spain), Bilbao (Basque Country, North Spain), Badajoz (Extremadura, Southwest Spain), and Madrid (Central Spain).

The study was approved by the Bioethics and Animal Well-being Committee of Instituto de Salud Carlos III, Majadahonda, Madrid, Spain. Written informed consent was obtained from all participants in the study.

### RNA extraction, RT-PCR amplification and sequencing

RNA was extracted using Nuclisens EasyMAG kit (bioMérieux, Marcy l’Etoile, France) from 1 ml of plasma, following manufacturer’s instructions. The HIV-1 protease-reverse transcriptase (PR-RT) *pol* segment and the C2-V3-C3 *env* segment were amplified by RT-PCR followed by nested PCR. Primers for PR-RT amplification were (sequences and HXB2 positions are indicated) RP1-S (GAAAAAGGGCTGTTGGAAATGTGGAA, 2016–2041) and RP-1-A (AAATTTAGGAGTCTTTCCCCATATTACTATGC, 3685–3716) in RT-PCR, and PR-O-S2 (GCTAATTTTTTAGGGAARATYTGGCCTT, 2080–2107) and RT-O-A (TGCCTCTGTTAATTGTTTTACATCATTAGTGTG, 3630–3662) in nested PCR, and those used for amplification of the V3 region were described previously [22]. Near full-length genome amplification was done in four overlapping segments, as described [23,24], using RNA extracted either from plasma or from the primary isolate’s culture supernatant grown from plasma using a previously described protocol [25].

Sequence electropherograms were viewed and assembled with Seqman (DNASTAR, Madison, WI, USA). For amplicons from suspected dual B/F1 infections, as indicated by frequent mixed peaks in sequence electropherograms at positions of B/F1 discordance, or, in one case, by sequences of different subtypes depending on sequencing primers used, TA cloning of the PCR products with subsequent clone sequencing was performed. In one sample with suspected dual B/F1 infection, nested PCR with PR-RT F1-specific primers (whose 3’ ends coincide with positions of B/F1 discordance) was performed. Sequences and HXB2 positions of these primers were PR-RT-nested-F1-S, GAAAAGAAGGACACCAAATGAAAGAATGC (2039–2067) and PR-RT-nested-F1-A, GTTAATTGTTTTACATCATTAGTGTGGGCAG (3625–3655).

Newly obtained sequences are deposited in GenBank under accessions KJ883030-KJ883089, KJ883091-KJ883108, KJ883110-KJ883152 and KT982428-KT982463.

### Phylogenetic sequence analyses

Sequences were aligned with MAFFT v.7 [26]. Phylogenetic trees were constructed via maximum likelihood with RAxML v.7.2.7 [27], applying the general time reversible substitution model with CAT approximation for among-site rate heterogeneity, with assessment of node support by bootstrapping. The possibility of intersubtype recombination was analyzed by bootscanning using Simplot v3.5 [28].

To identify viruses from other geographical areas available in public databases related to the subtype F cluster, we used BLAST searches followed by phylogenetic analyses. For this, we downloaded all HIV-1 F1 subsubtype sequences at the Los Alamos HIV Sequence Database [29] (n = 3,660) and examined similarity to all available F1 subsubtype near full-length genome sequences (n = 39), including the 17 obtained in this study, with local BLAST searches using BioEdit v.7.1.3.0 (Tom Hall, www.mbio.ncsu.edu/BioEdit/bioedit.html). Database sequences with the highest similarity scores to ≥2 viruses of the subtype F cluster were selected for phylogenetic analysis with RAxML.

### Antiretroviral drug resistance determination

Antiretroviral (ARV) drug resistance in PR-RT sequences was analyzed with the Calibrated Population Resistance Tool [30].

### Phylodynamic and phylogeographic analyses

To estimate emergence times of the most recent common ancestors (tMRCA) of clades and their geographical locations, and to analyze the demographic growth of the subtype F cluster, we used a Bayesian Markov Chain Monte Carlo (MCMC) coalescent method as implemented in BEAST v1.7.5 [31]. For this analysis, we used all PR-RT sequences of the subtype F cluster ≥1 kb. Since the evolutionary rate could not be inferred directly from the sequences, due to the narrow time span of sample collection, we estimated it from 68 F1 subsubtype PR-RT sequences from viruses collected along 22 years retrieved from the HIV Sequence Database or obtained by us [21]. Estimated substitution rates were then used as prior means (normal distribution) for the analysis of the subtype F cluster. We chose an HKY substitution model with gamma-distributed among-site rate heterogeneity and two partitions in codon positions (1st+2nd; 3rd) [32]; we also used an uncorrelated lognormal relaxed clock model and a Bayesian skyline plot demographic model [33]. Each MCMC chain was run for 150 million generations, sampling every 5,000. MCMC convergence and effective sample sizes (ESS) were checked with Tracer v.1.5 (http://tree.bio.ed.ac.uk/software/tracer/), ensuring that the ESS of each parameter was >200. Results were summarized with a maximum clade credibility (MCC) tree, using TreeAnnotator v1.5.3, after removal of a 50% burn-in. The MCC tree was visualized with FigTree v1.3.1. (http://tree.bio.ed.ac.uk/software/figtree/). Parameter uncertainty was summarized in the 95% highest posterior density (HPD) intervals.

## Results

**Fig 1:**
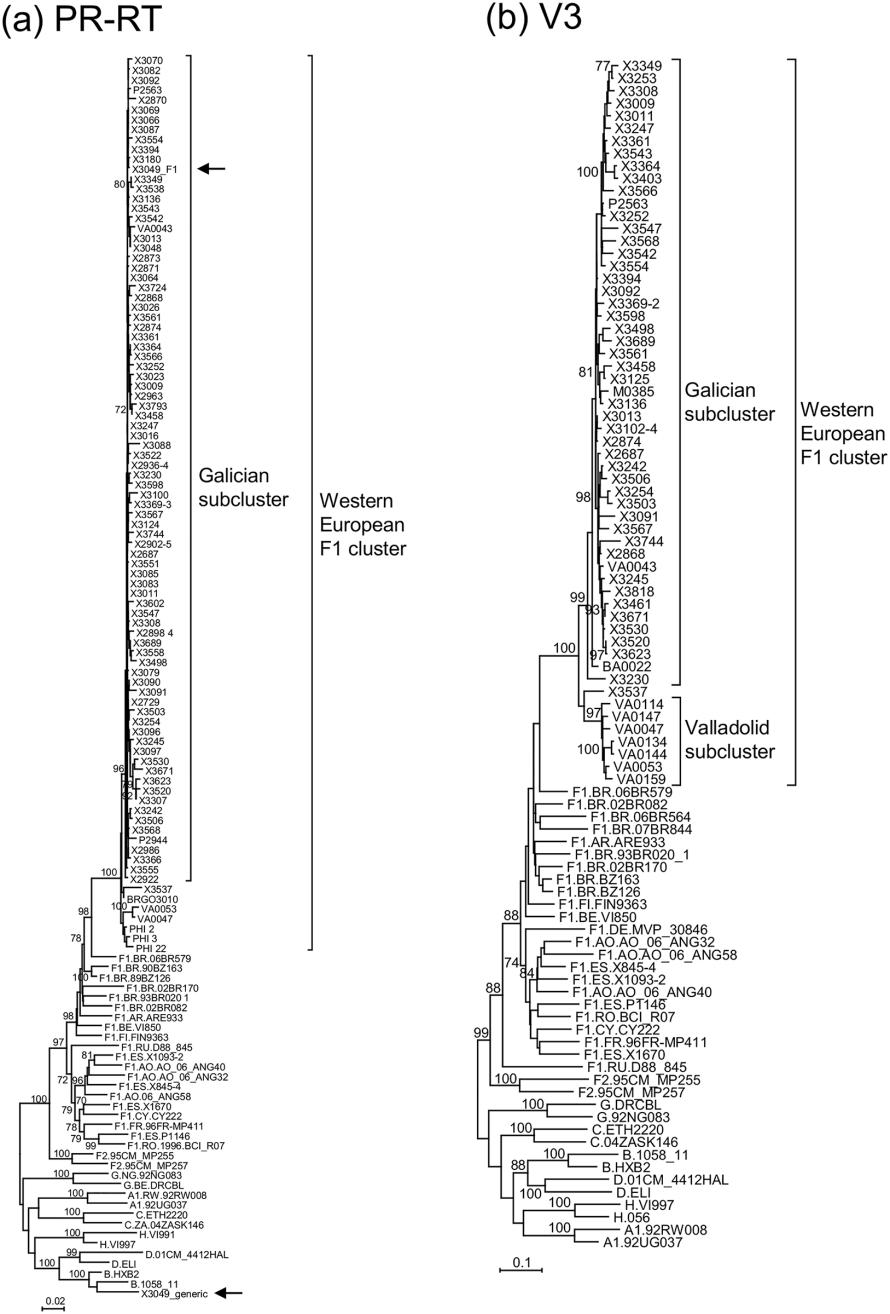
Maximum likelihood trees for the PR-RT (a) and V3 (b) segments of viruses of the subtype F cluster. The PR-RT tree, in addition to the sequences from Spain, also includes three sequences from Lausanne, Switzerland (PHI_2, PHI_3, PHI_22) and one from Goiania, Brazil (BRGO3010) previously reported by us to be closely related to the Spanish viruses of the subtype F cluster [21]. In Fig 1(a), both sequences derived from the X3049 sample (one obtained with generic primers and the other with F1-specific primers) (see Methods for details) are signaled with arrows. Both trees include F1 subsubtype references corresponding to viruses with near full-length genome sequences available in databases. Country of sample collection of these viruses is indicated with the ISO two-letter country code. Among viruses of this study collected in Spain, those with names starting with X, P, VA, BA, and M are from Galicia, Bilbao, Valladolid, Badajoz, and Madrid, respectively. Only bootstrap values ≥70% are shown.

Among HIV-1 infections diagnosed in Spain in 2009–2013, we identified 87 viruses of subtype F, mainly from Galicia, that formed a clade for the PR-RT sequence (Fig 1a). None of the 1,660 HIV-1 samples collected during 1999–2008 in Galicia and previously sequenced by us branched in this cluster. Three viruses of the subtype F cluster (one from Galicia and two from Valladolid, in central Spain) branched off basally to the rest, which formed a subcluster. We will refer to this subcluster as the Galician subcluster. Similarly, we will refer to the entire subtype F cluster, comprising viruses from Spain and other Western European countries and one from Brazil [identified in a previous [21] or in this study (see below)] as the Western European subtype F cluster. For one virus from Galicia, X3049, whose PR-RT bulk sequence was of subtype B (Fig 1a), the sequence electropherogram showed numerous mixed positions with minor peaks overlapping major peaks, most of them corresponding to positions where B subtype and F cluster consensuses differ. Suspecting dual B/F1 infection, we obtained 19 clones for the amplified PR-RT fragment, all of which were of subtype B; however, nested PCR with F1 subsubtype-specific primers yielded an amplicon whose sequence branched within the Galician F subcluster (Fig 1a), indicating the presence of a dual B subtype/F cluster infection. Another virus from Galicia, X3461, also showed numerous mixed electropherogram peaks only in protease at positions differing between B subtype and the F cluster. Nineteen PR-RT clones of this virus, analyzed by bootscanning, revealed an infection with diverse variants, with 18 clones being BF1 recombinant and one of subtype B (S1 Fig). Most recombinant clones had 5’-F/B-3’ structures, with breakpoints near the protease-RT junction in 12 and at around position 120 of protease in 7, and one had a B/F/B structure, with breakpoints at around positions 120 and 290 of protease (S1 Fig); the subtype B fragment of the recombinant clones derived from the subtype B strain represented by a single clone (S1 and S2 Figs). A phylogenetic tree of protease of the recombinant clones of X3461 having the largest subtype F fragment showed branching in the subtype F cluster (S3 Fig). Three additional viruses from Galicia (X2890, X2955, and X3253) that could not be RT-PCR-amplified for PR-RT but were amplified for protease, also branched within the subtype F cluster for this segment (S3 Fig). Assignment of protease sequences to the Galician subcluster was uncertain because the subcluster was not well supported in the protease tree (S3 Fig). Analysis of ARV drug resistance mutations only revealed a minor population with protease M46I, associated with low level resistance to Nelfinavir, in a patient who was under treatment with atazanavir/ritonavir.

In phylogenetic analyses of the *env* V3 region of viruses from Spain, we identified 59 sequences that branched within the subtype F cluster, 48 corresponding to viruses also analyzed for PR-RT or protease, and 11 to viruses only sequenced for V3. In the V3 tree, 8 viruses (7 from Valladolid and 1 from Galicia) fell outside the Galician subcluster, with the viruses from Valladolid forming a well-supported subcluster (Fig 1b). The V3 region sequence of X3461 was of subtype F or B depending on the primers (forward or reverse) used for sequencing. Therefore, we performed TA cloning of the PCR products with subsequent clone sequencing; this allowed us to confirm the dual nature of the infection in the V3 region of X3461, with some clones branching with viruses of the subtype F cluster and others with subtype B references (S4 Fig). The subtype F V3 sequences of X3461 and that of X3253 fell in the Galician subcluster. In total, 100 viruses of the subtype F cluster were identified in Spain, of which 90 corresponded to the Galician subcluster.

**Fig 2:**
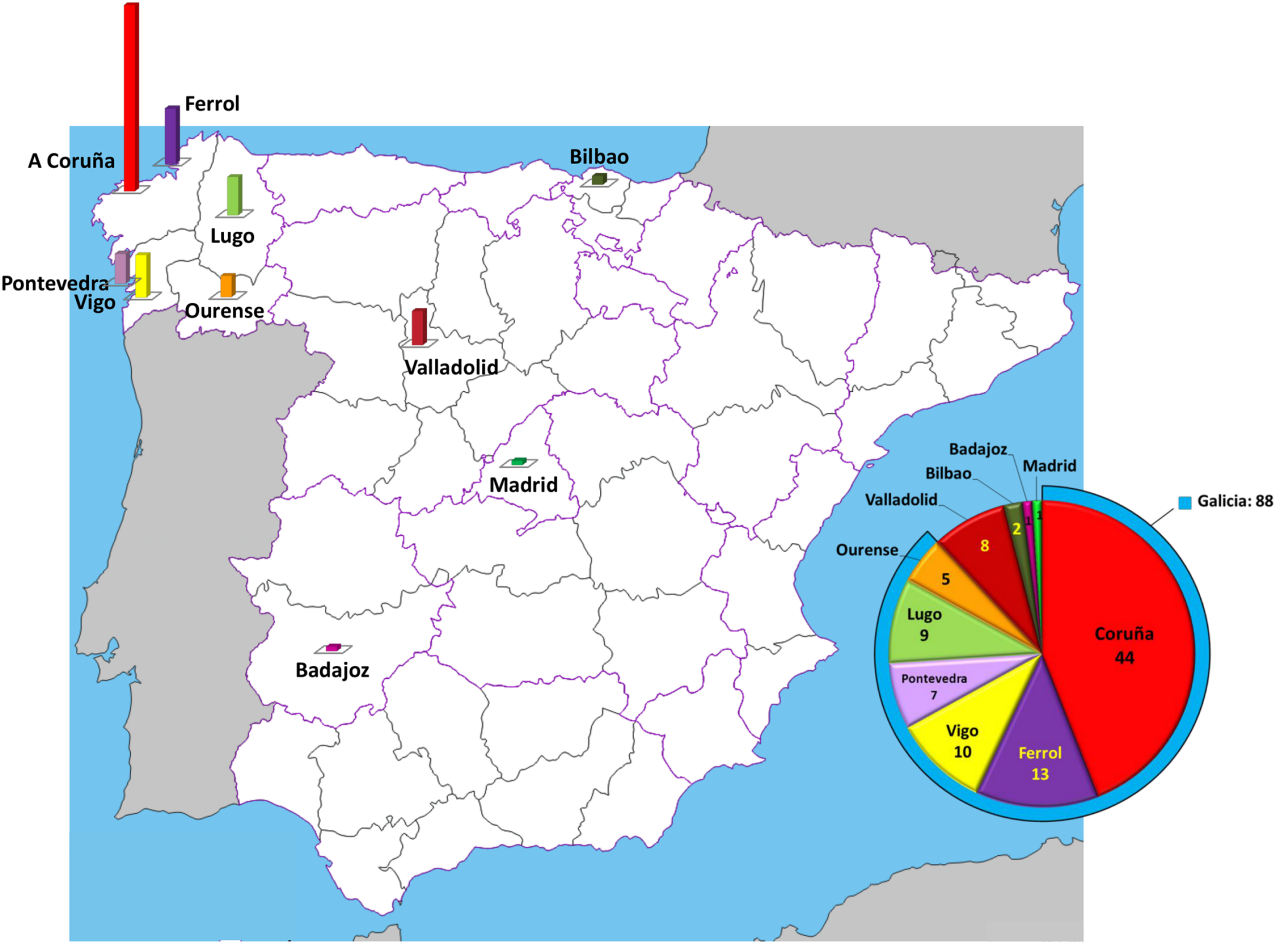
Distribution of viruses of the subtype F cluster sequenced by us according to city of sample collection.

Of the 100 viruses of the subtype F cluster sequenced by us, 88 were collected in Galicia (44 in the city of A Coruña), 8 in Valladolid, 2 in Bilbao, 1 in Badajoz, and 1 in Madrid (Fig 2). All individuals harboring them were men, and all 94 with reported transmission routes were infected sexually. Of these, at least 71 were MSM, according to their self-reported sexual behavior, although 11 were reportedly heterosexual, and for 12 the sexual transmission mode was not specified. The countries of origin, reported for 91 individuals, were Spain (n = 80), a Latin American country (n = 9), Israel (n = 1), and Equatorial Guinea (n = 1). At least 97 samples were from new HIV-1 diagnoses and at least 12 were from recent seroconversions (less than one year from last seronegative to first seropositive samples).

**Fig 3:**
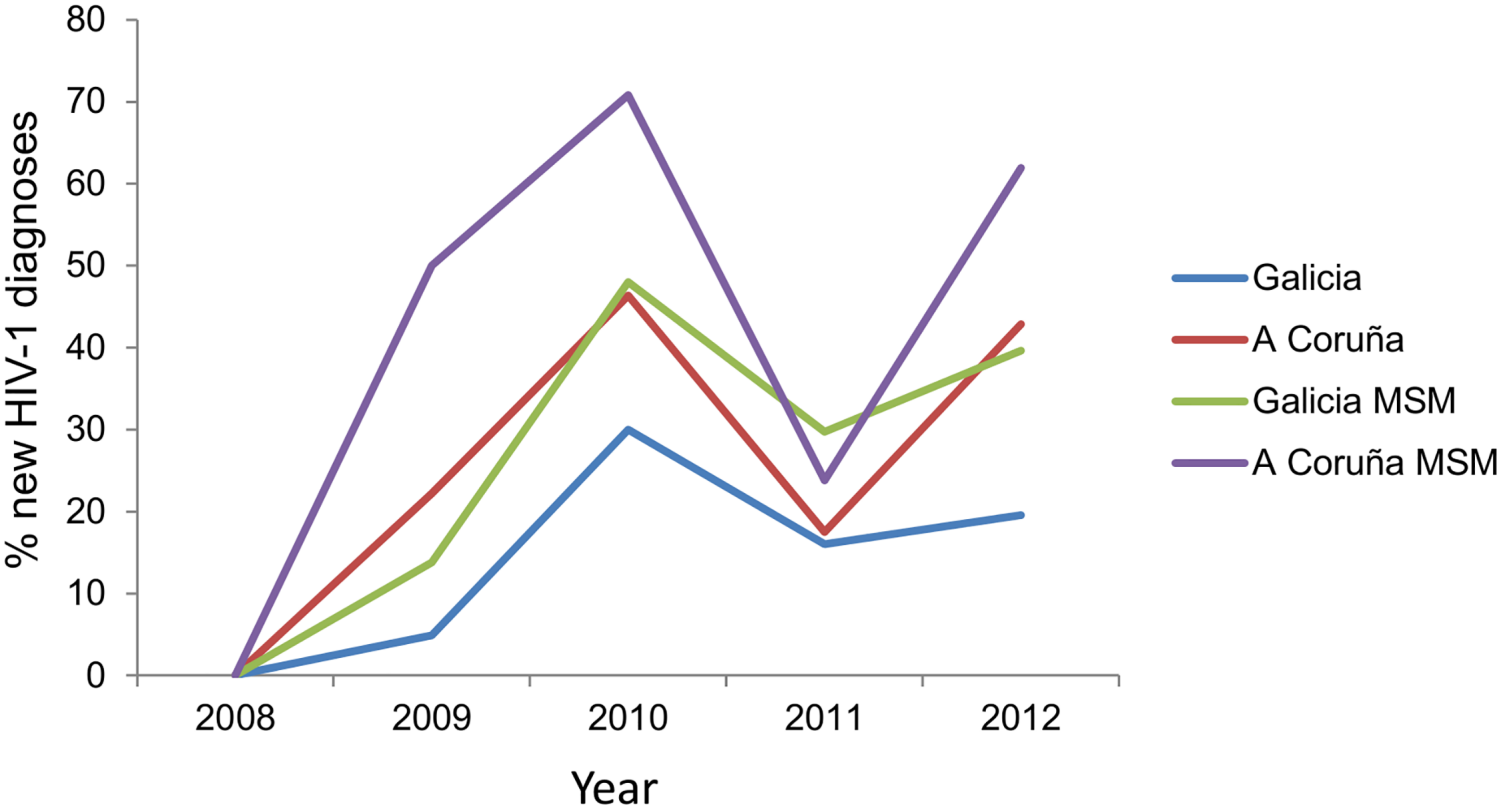
Prevalence of infections with viruses of the subtype F cluster in 2009–2012 among all new HIV-1 diagnoses and among new HIV-1 diagnoses in MSM in Galicia and A Coruña city (Northwest Galicia).

Percentages of newly diagnosed HIV-1 infections caused by viruses of the subtype F cluster in Galicia and in the city of A Coruña, where the greatest number of infections of this cluster was diagnosed, are shown in Fig 3. From 2009 to 2012 percentages in Galicia were 4.9%, 27.3%, 14.2%, and 19.6%, respectively; while in A Coruña they were 22.2%, 46.3%, 17.5%, and 42.9%, respectively. Considering only MSM, percentages in Galicia were 13.8%, 46%, 24.3%, and 39.6%, respectively; while in A Coruña they were 50%, 70.8%, 23.8%, and 61.9%, respectively. (Data from 2013 were not included because samples from A Coruña diagnosed that year were not available for sequencing.)

**Fig 4:**
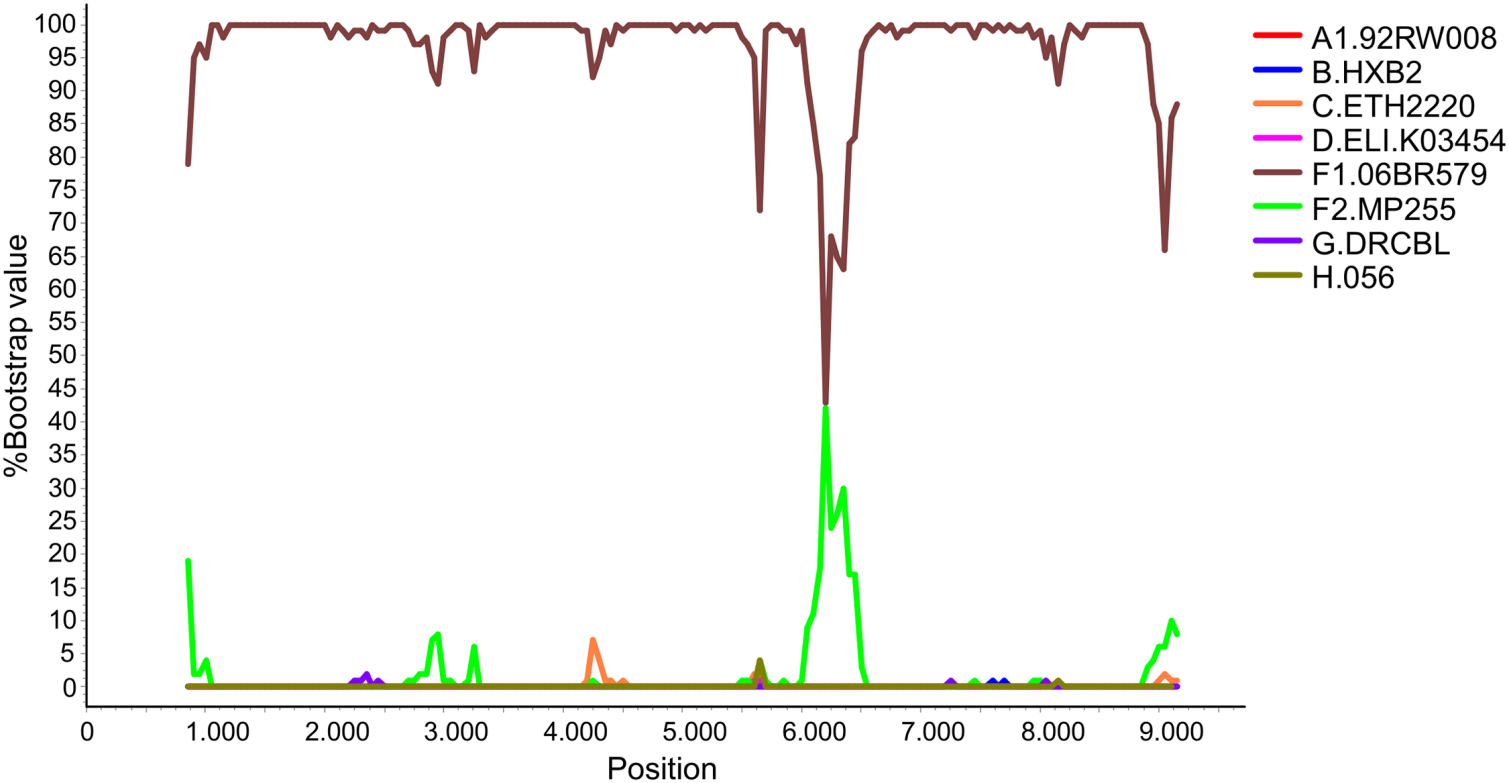
Bootscan analysis of the near full-length genome sequence of X3364. The analysis was done with Simplot v.3.5.1, using a window of 500 nucleotides, moving in 50 nucleotide increments. Phylogenetic trees were constructed using the neighbor-joining algorithm based on Kimura 2-parameter distances, with Tv:Ti ratios estimated from the dataset.

**Fig 5:**
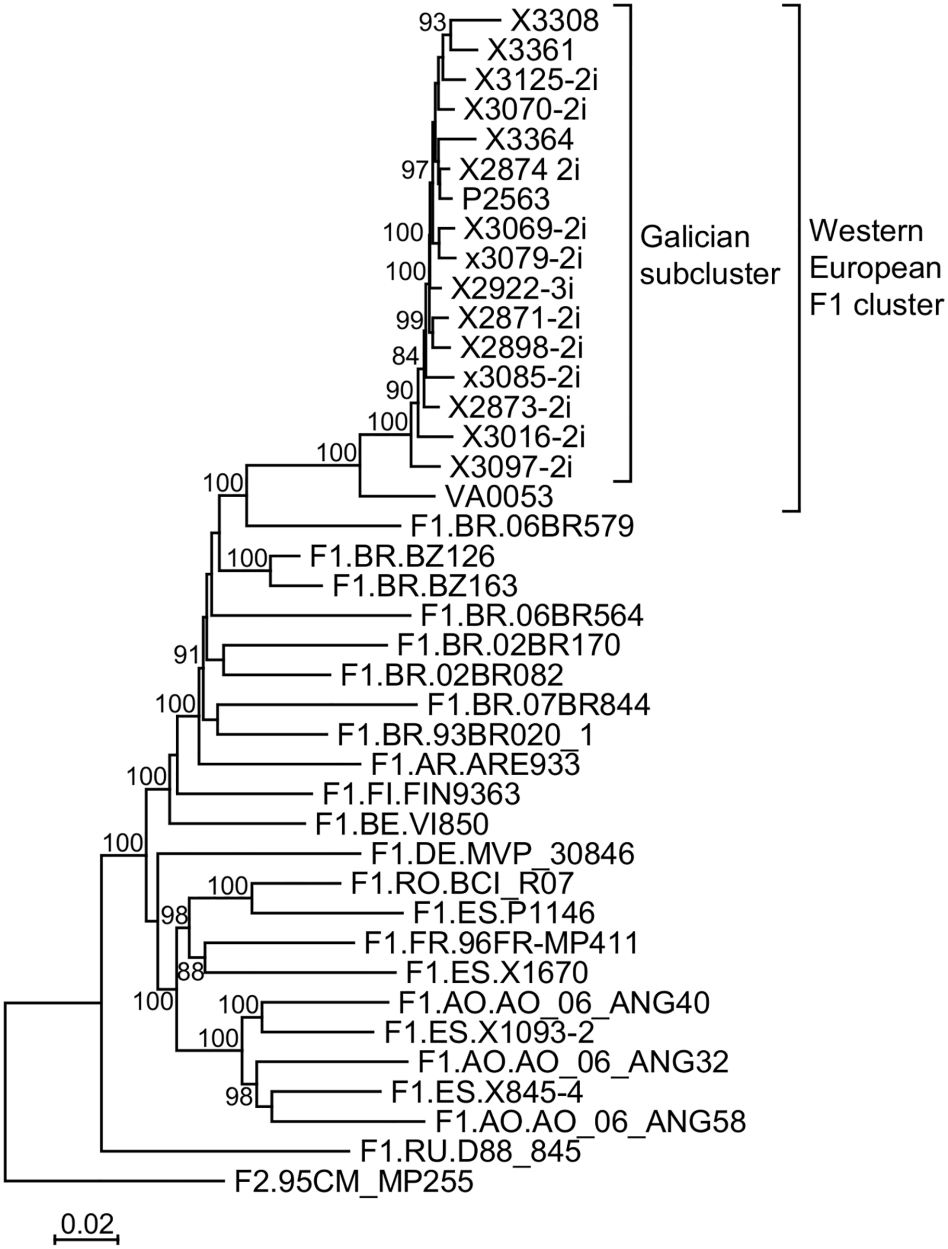
Maximum likelihood tree of near full-length genome sequences of viruses of the subtype F cluster. F1 subsubtype references are included in the analysis, with country of sample collection indicated with the ISO two-letter country code. The tree is rooted with an F2 subsubtype sequence. Only bootstrap values ≥80% are shown.

We obtained near full-length genome sequences for 17 viruses of the subtype F cluster. All were uniformly of F1 subsubtype in the bootscan analyses (Fig 4). In the phylogenetic tree (Fig 5) they formed a cluster, with the virus from Valladolid branching in a basal position and the virus 06BR579, from Sao Paulo, Brazil, as its closest relative.

**Fig 6:**
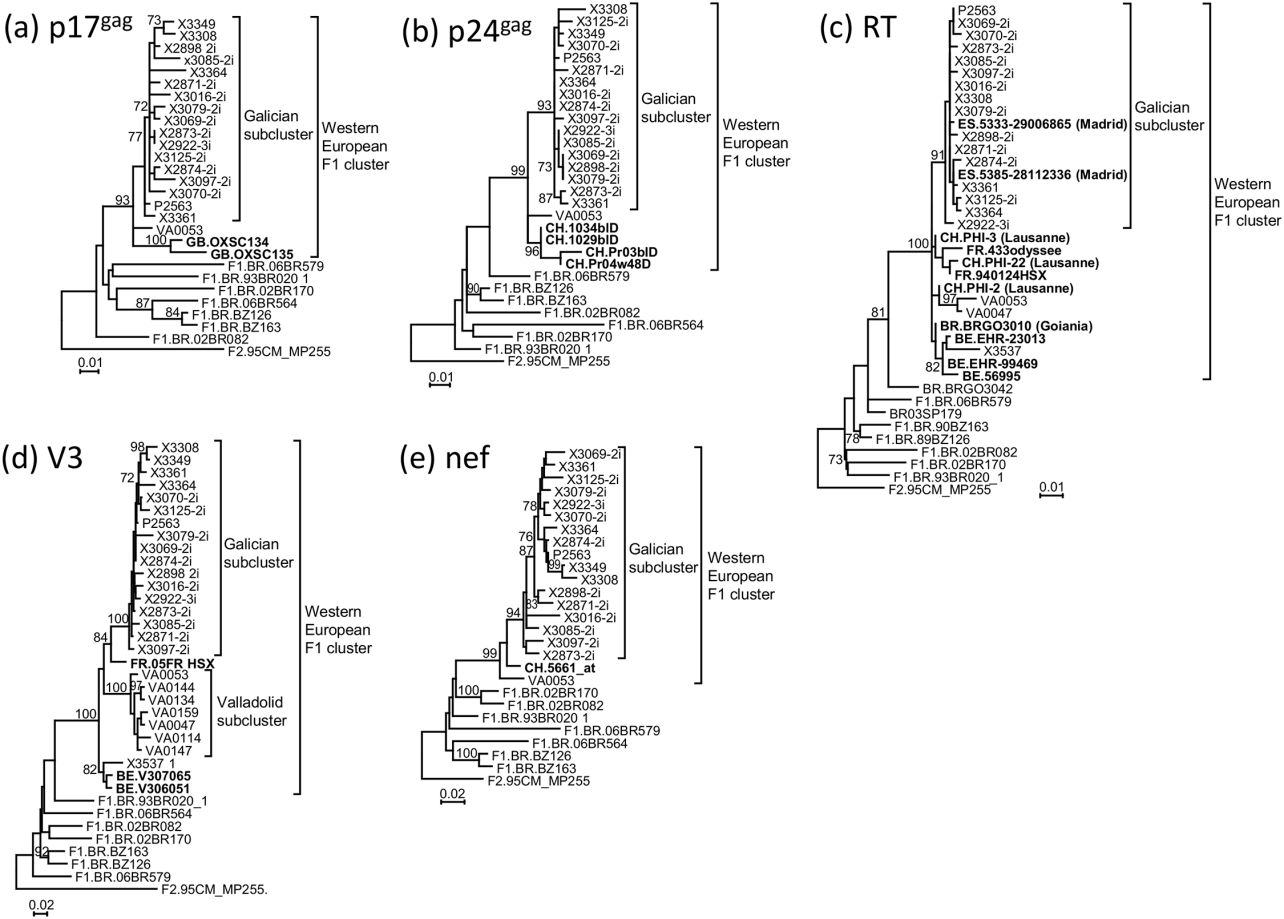
Maximum likelihood trees of sequences from databases belonging to the Western European subtype F cluster. The trees correspond to genome segments in (a) p17^gag^ (HXB2 positions 790–1185), (b) p24^gag^ (HXB2 positions 1575–2042), (c) reverse transcriptase (HXB2 positions 2637–3278), (d) *env* V3 region (HXB2 positions 6709–7448), and (e) *nef* (HXB2 positions 8797–9417). References for the subtype F cluster are near full-length genome sequences obtained by us, with names beginning with X, P or VA, with ‘i’ ending denoting those derived from cultured isolates. Database viruses branching in the subtype F cluster are in bold type. Countries of sample collection of database viruses are indicated with the ISO two-letter codes: BE, Belgium; BR: Brazil; CH, Switzerland; ES, Spain; FR, France; GB, United Kingdom. Cities of sample collection of database viruses of the subtype F cluster, when known, are in parentheses. Only bootstrap values ≥70% are shown.

Through local BLAST searches with all F1 subsubtype viruses downloaded from the Los Alamos Database, using all available near full-length genome F1 susbsubtype sequences as references, followed by phylogenetic analyses, we identified 21 partially-sequenced database viruses branching within the Western European subtype F cluster, which included 5 previously reported by us [21]. Of those viruses, 2 were collected in Madrid [34], 8 in Switzerland [35], 5 in Belgium [36], 3 in France [12], 2 in the United Kingdom (UK), and 1 in Goiania, West-Central Brazil [37] (Fig 6). A second virus from Goiania (BRGO3042) was related to the Western European cluster, branching outside of it (Fig 6c). Both viruses from Madrid branched within the Galician subcluster, and all non-Spanish viruses branched outside of both Galician and Valladolid subclusters. All 4 Swiss viruses analyzed for p24^gag^ grouped in a subcluster (Fig 6b). Similarly, three viruses from Belgium and one from Galicia (X3537) analyzed for RT grouped in a subcluster (Fig 6c). The common ancestry of X3537 and the Belgian viruses was also supported for the V3 region (Fig 6d). According to the information available in databases or in published studies [12,35], at least 9 infections with non-Spanish viruses of the Western European subtype F cluster correspond to primary or recent infections. Data on transmission routes were available for 5 viruses, 3 from Switzerland [35] and 2 from Belgium [36]; all were sexually transmitted, with 4 corresponding to MSM. Sample collection dates ranged from 2005 to 2011, with the earliest dates in 2005 corresponding to 4 viruses from Switzerland.

**Fig 7:**
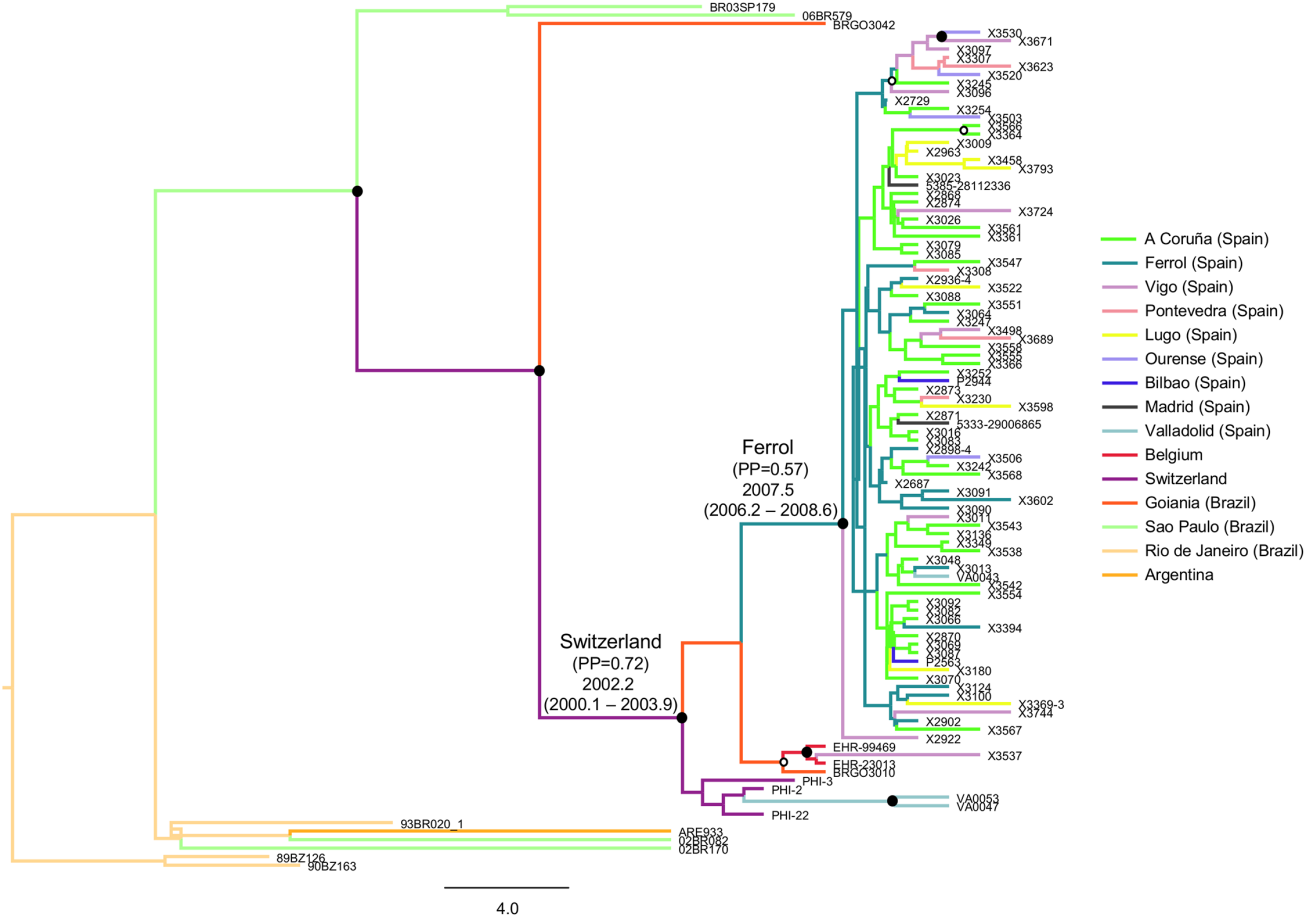
Maximum clade credibility tree of PR-RT sequences of the subtype F Western European cluster and Galician subcluster. Nodes supported by PP = 1 and PP = 0.95–0.99 are marked with filled and unfilled circles, respectively. Colors of terminal and internal branches represent sampling locations and most probable locations of the corresponding nodes, respectively, according to the legend on the right. For the nodes corresponding to the Galician subcluster and the Western European cluster, the posterior probabilities for the most probable locations and the tMRCAs are indicated above the subtending branches (95% HPD intervals are in parentheses).

To estimate the origins of the Western European cluster and of the Galician subcluster, we analyzed, using a Bayesian approach, PR-RT sequences of the Western European subtype F cluster, which, in addition to the Galician viruses, included viruses from Valladolid (n = 3), Madrid (n = 2), Bilbao (n = 2), Switzerland (n = 3), Belgium (n = 2), and Goiania, Brazil (n = 1). We also included all F1 subsubtype viruses of the Brazilian variant with available near full-length sequences lacking drug resistance-associated mutations, and two other viruses from Brazil (BR03SP179, from Sao Paulo, and BRGO3042, from Goiania) which were found to be related to the Western European subtype F cluster, branching outside of it, in phylogenetic analyses (Fig 6c). The results, summarized in the MCC tree (Fig 7), estimated the tMRCA of the Galician subcluster in 2007.5 (95% HPD 2006.2–2008.6) and that of the Western European cluster in 2002.2 (95% HPD 2000.1–2003.9). The most probable origin of the Galician subcluster was estimated in Ferrol [0.57 posterior probability (PP)], followed by A Coruña (distant 20 km by sea and 55 km by road from Ferrol) (PP = 0.16). The most probable origin of the Western European cluster was Switzerland (PP = 0.72) followed by Goiania (PP = 0.1). Since Ferrol and A Coruña are geographically close, as are Vigo and Pontevedra (distant 30 km), we repeated the Bayesian analysis combining the samples from Ferrol and A Coruña and those from Vigo and Pontevedra. In this new analysis, the most probable location for the origin of the Galician subcluster was Ferrol-Coruña (PP = 0.88).

**Fig 8:**
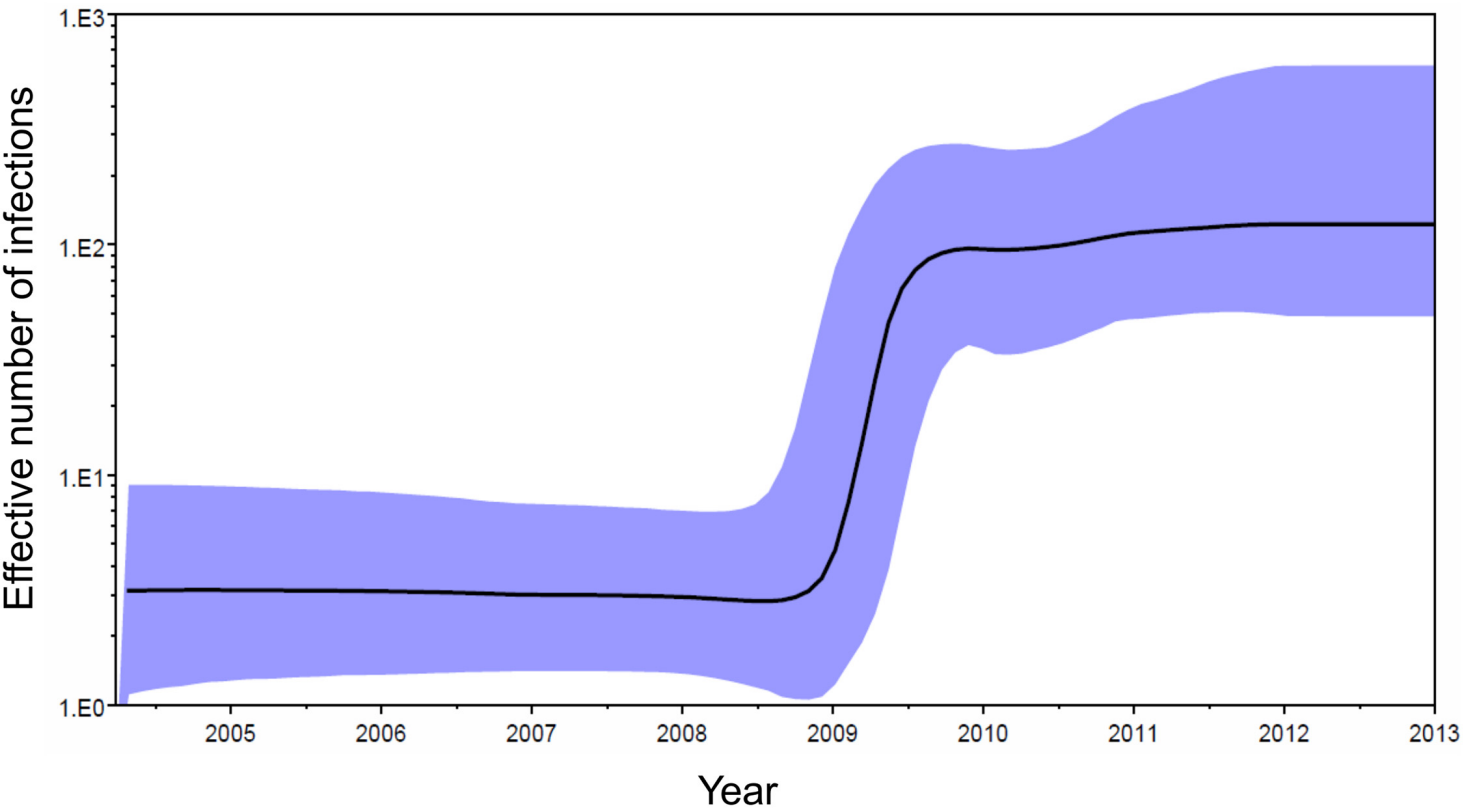
Bayesian skyline plot of the population growth of the subtype F cluster. The black line represents the median estimate of the effective number of infections through time (logarithmic scale) and the shaded area represents the 95% HPD credibility interval. The horizontal axis represents calendar years.

The Bayesian skyline plot (Fig 8) showed an exponential growth in the effective number of infections starting at the end of 2008 and continuing through the first half of 2009, with slower growth rates in 2010 and 2011 and stabilization in 2012.

## Discussion

The most remarkable finding of this study is probably that the Galician HIV-1 subtype F cluster previously reported by us [21] is part of a larger cluster that has independently spread in at least three other Western European geographic areas, as evidenced by local clustering of sequences from Valladolid, central Spain (7 infections) (Fig 1b), Switzerland (7 infections) (Fig 5b and 5c) (although clustering of 3 Swiss viruses is not strongly supported in PR-RT, they share K103N drug resistance mutation [35], and in p24^gag^ the 4 Swiss viruses group with a 96% bootstrap value), and Belgium (5 infections, with a sixth virus from Galicia clustering with them) (Fig 6c and 6d). Additionally, 2 newly diagnosed infections from the UK branching within the Western European subtype F cluster are closely related to each other (Fig 6a), and 3 viruses from France, with uncertain mutual relations, also branch within the Western European subtype F cluster (Fig 6c and 6d). The fact that most of the viruses are from recent diagnoses, with 7 infections from Switzerland [35] and 2 from France [12] being from primary or recent infections, also indicates that viruses of the Western European subtype F cluster are propagating throughout multiple local networks. The available data indicate that transmission takes place among MSM, since all Spanish cases studied by us are sexually-infected men, at least 71 MSM, as are 3 infections from Switzerland (2 from MSM) [35] and 2 from Belgium (both from MSM) [36]. Considering that all 100 infections of the cluster studied by us were detected in men, we strongly suspect that those cases from Spain reported to be transmitted via heterosexual or unspecified sexual contact also correspond to MSM.

Since our initial report [21] comprising samples collected up to April 2011, the number of viruses of the Galician subtype F subcluster identified by us has increased from 38 individuals to 90, including individuals diagnosed outside of Galicia in the cities of Bilbao (n = 2), Valladolid (n = 1), Badajoz (n = 1), and Madrid (n = 1). It is important to note that this study does not derive from a national survey. Outside of Galicia, we have only analyzed samples from all public hospitals in the Basque Country and in the cities of Valladolid, Badajoz, León, and Toledo, and from two hospitals in the province of Madrid. Therefore, it is most likely that the real size of the subtype F cluster in Spain is considerably larger than here described. Comparing with other non-subtype B clusters reported in Western Europe [7,18–20,38–40], the Galician subtype F subcluster is substantially larger than all of them, followed by a CRF01_AE cluster comprising 46 injecting drug users reported in Sweden [40], and is part of an even larger cluster comprising viruses from other regions of Spain and at least four other Western European countries and Brazil. In a recent study [41] analyzing HIV-1 transmission networks using 84,757 PR-RT sequences available at the Los Alamos HIV Sequence Database, with clustering based on genetic distances ≤1%, the originally described Galician subtype F cluster [21] represented the largest HIV-1 network in Western Europe and one of the largest in the world.

Using a Bayesian phylogeographic approach, the most probable origin of the Galician subtype F subcluster was estimated in Ferrol, province of A Coruña, Northwest Spain (PP = 0.57), with the second most probable location being the city of A Coruña, near Ferrol (PP = 0.16) (Fig 7). This is consistent with the places of the earliest diagnosed infections with viruses of the Galician subcluster studied by us: the first two cases were diagnosed in Ferrol in March and May 2009 and the next six in A Coruña, two in November-December 2009 and four in January-March 2010. Our Bayesian analysis placed the origin of the Western European subtype F cluster in Switzerland (PP = 0.72). This is also consistent with the years of sample collection, which among infections diagnosed in Switzerland were 2005 for four and 2006 for one, all from primary infections [35], while for Belgian infections they were 2006 through 2010, and for the Goianian infection it was 2007. The spread of the subtype F cluster in Valladolid appears to be more recent, with no diagnoses dating earlier than 2011. It is important to note that the conclusions drawn from the phylogeographic analyses about the ancestral locations of clades should be interpreted with caution, since geographic sampling is incomplete. However, we are confident that the Galician subtype F outbreak probably originated in Ferrol, since we have analyzed most of the new HIV-1 diagnoses from Galicia. In contrast, the estimated origin of the Western European cluster (Switzerland) is less certain, considering the relative paucity of available HIV-1 sequences from new infections.

The tMRCA estimated for the Galician subtype F subcluster was around 2007, which is consistent with the date previously estimated by us using a smaller dataset [21], but differs widely from a younger estimate (1993), recently proposed by other authors based on samples from two cities in Galicia [42]. The sequences used in the latter study are currently unavailable in public databases. However we find difficult to reconcile the 1993 estimate with (1) the total absence of viruses of the Western European subtype F cluster in our surveillance analyses of 1,660 HIV-1 samples collected across Galicia from 1999 to 2008; and with (2) the date (2002) and geographical origin (Switzerland) estimated here for this cluster using all available sequences. It is possible either that the basal sequence presumably responsible for the estimated tMRCA in [42] may not represent the origin of the Western European subtype F cluster, but rather had a separate ancestry in a Brazilian subtype F variant related to the MRCA of the mentioned cluster; or that the mentioned sequence is of recombinant origin, since recombinant sequences have a tendency to branch basally to one of the parental clades, mimicking an ancestral split [43].

The rapid spread of the subtype F cluster in Galicia, where its prevalence among new HIV-1 diagnoses grew from 0% in 2008 to 27% (46% among MSM) in 2010, is remarkable (Fig 3). The increase was even more prominent in the city of A Coruña, where the percentage reached 46% (71% among MSM) in 2010. The rapid growth of the subtype F cluster is also supported by the Bayesian skyline plot analysis, which estimates that most of its growth occurred in less than one year from the end of 2008 (Fig 8). Its rapid spread in Galicia, together with its propagation in multiple geographically distant locations, suggests the existence of some inherent biological property of the viruses of this cluster favoring transmission. It is difficult to ascribe its rapid growth only to unusually risky and promiscuous sexual behaviors among a group of MSM in Galicia, since we have detected multiple other clusters among MSM in this region and none of them has approached the growth dynamics of the subtype F cluster. The second largest cluster detected in Galicia comprises 44 subtype B viruses, most of them from MSM, diagnosed during an 8 year period (compared to 90 in less than 5 years for the Galician subtype F subcluster). At least 3 individuals of the subtype F cluster had dual infections (2 detected initially and 1 during the follow-up), and in 2 of them the second virus belonged to a Galician subtype B cluster, one of them being the largest of subtype B in this region. This suggests that the subtype F network overlaps with other sexual networks in Galicia, which makes difficult to explain solely on epidemiological grounds why no other clusters circulating in Galicia have spread at a similar speed as the subtype F cluster. Moreover, its spread in local networks in at least four distant areas from three countries, a phenomenon unreported for other Western European HIV-1 clusters, also supports the idea of an unusual high transmissibility of the subtype F cluster.

In summary, the HIV-1 subtype F cluster here described is the largest and most widely geographically spread nonsubytpe B cluster reported in Western Europe, exhibiting an unusually rapid expansion and involving multiple separate sexual networks in distant geographical areas. These epidemiological features suggest some inherent biological properties favoring sexual transmission, whose study will require further work. In this regard, distinct *in vivo* biological features of viruses of the Galician subtype F cluster have been reported recently, with significantly higher plasma viral loads and poorer virologic responses to antiretroviral therapy compared to subtype B viruses [44]. The rapid expansion of this cluster in Galicia is reminiscent of that of other genetic forms in established HIV-1 epidemics, such as subtype C in southern Brazil [45], BG recombinant viruses in Cuba [46], subtype A in Greece [47], CRF01_AE among MSM in China [48], or CRF63_02A1 in Siberia [49]. This highlights the need for continued molecular epidemiological surveillance of HIV-1 variants, which may have multiple implications, including the design of vaccine immunogens [50,51]. Finally, it is important to note that the vast majority of infections of the subtype F cluster were newly diagnosed and that its rapid expansion in Galicia took place in the context of a public health care system covering all HIV-1-infected individuals. This indicates that the spread of the Galician subtype F cluster is mostly driven by transmission from recently infected individuals unaware of their HIV status, as reported for other clusters transmitted among MSM [3,4,8,10–13], and that, contrary to some beliefs, universal access to effective antiretroviral therapies may not result in substantial control of the epidemic spread of HIV-1 [2,52]. Therefore, it will be very important to implement public health measures aimed at reducing high risk sexual behaviors and to support efforts for the development of an effective vaccine and other methods for prevention of HIV-1 transmission in order to contain the epidemic.

## Supporting Information

S1 Fig Bootscan analyses of four PR-RT clones of X3461. The analysis was done using an window of 300 nucleotides for clone 18 and of 150 nucleotides for all other clones, moving in 20 nucleotide increments. Phylogenetic trees were constructed using the neighbor-joining algorithm based on Kimura 2-parameter distances, with Tv:Ti ratios estimated from the dataset. The analyses were done using subtype references, with a Galician F1 cluster virus used as F1 reference. For clones showing a BF recombinant structure, new bootscan analyses (shown in the figure) were done incorporating the subtype B clone 18 as reference strain. Eleven other clones had bootscan plots virtually identical to that of clone 7 and seven to that of clone 4.
(TIF)

S2 Fig Maximum likelihood tree for the RT gene of X3461 clones. Only bootstrap values ≥70% are shown.
(TIF)

S3 Fig Maximum likelihood tree of the protease gene for X2890, X2955, and X3253, and for 12 F^PR^/B^RT^ recombinant clones of X3461 (labeled with circles), analyzed together with representative viruses of the F1 cluster and F1 subsubtype references. Only bootstrap values ≥70% are shown.
(TIF)

S4 Fig Maximum likelihood tree of X3461 clones of the V3 region. Sequences obtained by bulk sequencing of the PCR product using forward [X3461_bulk(a)] or reverse [X3461_bulk(b)] primers are also included in the analysis. Only bootstrap values ≥70% are shown.
(TIF)
