# Supplementary figures and images for: Phylogeny and Phylogeography of a Recent HIV-1 Subtype F Outbreak among Men Who Have Sex with Men in Spain Deriving from a Cluster with a Wide Geographic Circulation in Western Europe
Source: PLoS One. 2015 Nov 24;10(11):e0143325. doi: 10.1371/journal.pone.0143325 (PMC4658047; doi:10.1371/journal.pone.0143325)

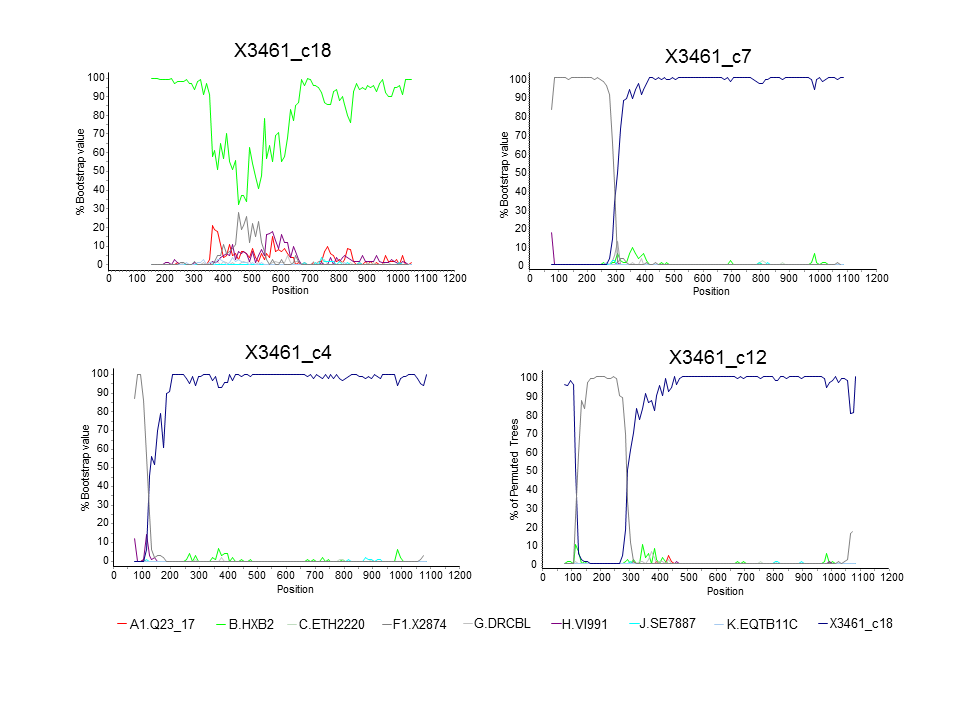

Supplement: S1 Fig — The analysis was done using an window of 300 nucleotides for clone 18 and of 150 nucleotides for all other clones, moving in 20 nucleotide increments. Phylogenetic trees were constructed using the neighbor-joining algorithm based on Kimura 2-parameter distances, with Tv:Ti ratios estimated from the dataset. The analyses were done using subtype references, with a Galician F1 cluster virus used as F1 reference. For clones showing a BF recombinant structure, new bootscan analyses (shown in the figure) were done incorporating the subtype B clone 18 as reference strain. Eleven other clones had bootscan plots virtually identical to that of clone 7 and seven to that of clone 4. (TIF) [file pone.0143325.s001.tif]

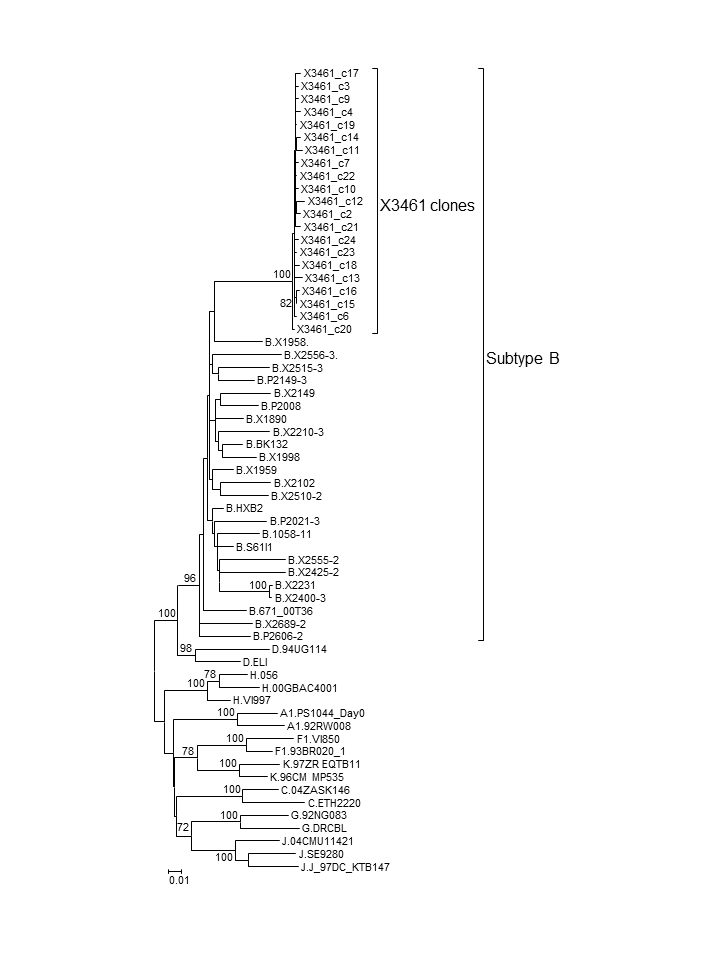

Supplement: S2 Fig — Only bootstrap values ≥70% are shown. (TIF) [file pone.0143325.s002.tif]

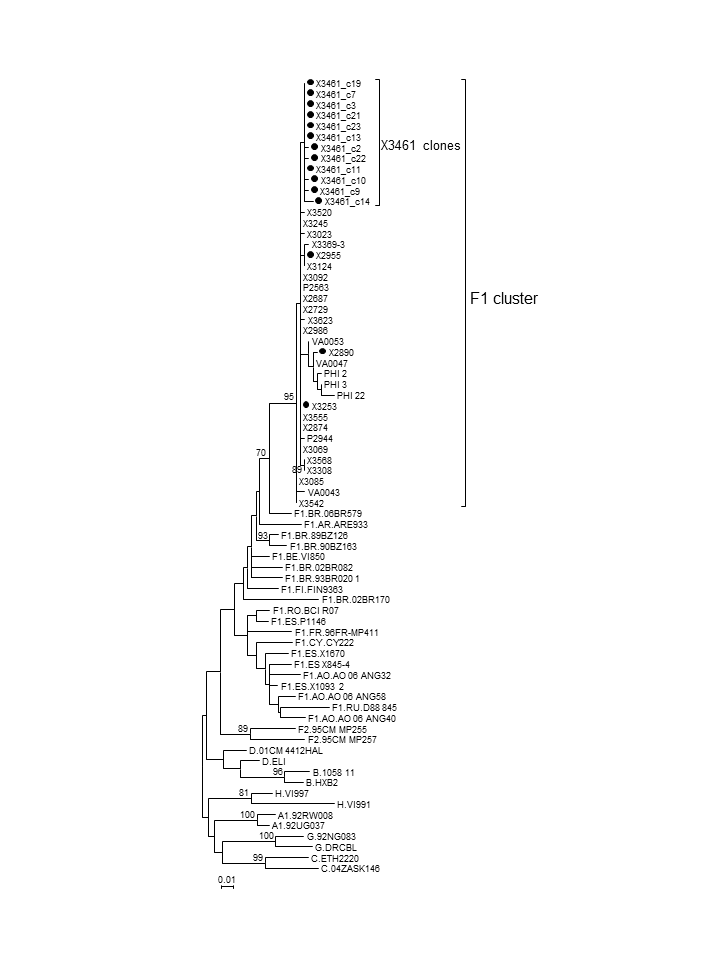

Supplement: S3 Fig — Only bootstrap values ≥70% are shown. (TIF) [file pone.0143325.s003.tif]

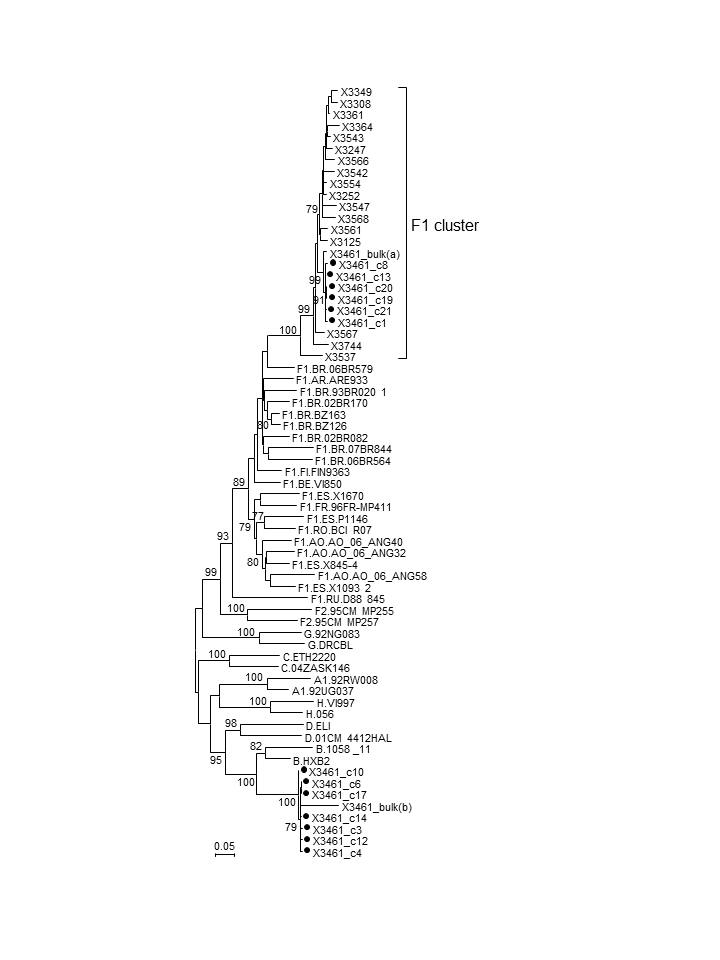

Supplement: S4 Fig — Sequences obtained by bulk sequencing of the PCR product using forward [X3461_bulk(a)] or reverse [X3461_bulk(b)] primers are also included in the analysis. Only bootstrap values ≥70% are shown. (TIF) [file pone.0143325.s004.tif]
